# Tranexamic Acid Administered During Off-Pump Coronary Artery Bypass Graft Surgeries Achieves Good Safety Effects and Hemostasis

**DOI:** 10.3389/fcvm.2022.775760

**Published:** 2022-02-04

**Authors:** Enshi Wang, Xin Yuan, Yang Wang, Weinan Chen, Xingtong Zhou, Shengshou Hu, Su Yuan

**Affiliations:** ^1^Department of Cardiovascular Surgery, Fuwai Hospital, Peking Union Medical College, Chinese Academy of Medical Sciences, Beijing, China; ^2^Medical Research and Biometrics Center, National Center for Cardiovascular Diseases, Beijing, China; ^3^Information Center, Fuwai Hospital, Peking Union Medical College, Chinese Academy of Medical Sciences, Beijing, China; ^4^Department of Anesthesiology, Fuwai Hospital, Peking Union Medical College, Chinese Academy of Medical Sciences, Beijing, China

**Keywords:** blood transfusion, off-pump coronary artery bypass grafting, risk analysis, thromboembolism, tranexamic acid, propensity score matching

## Abstract

**Background:**

Tranexamic acid (TXA) administered during off-pump coronary artery bypass (OPCAB) surgeries has achieved good blood control in small cohorts. We aimed to investigate the safety issues and hemostasis associated with TXA administration during OPCAB in a large retrospective cohort study.

**Methods:**

This study included 19,687 patients with OPCAB from 2009 to 2019. A total of 1,307 patients were excluded because they were younger than 18 years or certain values were missing. Among the remaining 18,380 patients, 10,969 were in the TXA group and 7,411 patients were in the no-TXA group. There were 4,889 patients whose TXA dose was ≥50 mg/kg, and the remaining 6,080 patients had a TXA dose of <50 mg/kg. Propensity score matching (PSM) was performed between the TXA and no-TXA groups and between the high-dose and low-dose groups, and statistical analysis was performed.

**Results:**

Tranexamic acid administration did not increase the risk of hospital death or thromboembolic events. Patients who administered TXA had less blood loss at 24 h (478.32 ± 276.41 vs. 641.28 ± 295.09, *p* < 0.001) and 48 h (730.59 ± 358.55 vs. 915.24 ± 390.13, *p* < 0.001) and total blood loss (989.00 ± 680.43 vs. 1,220.01 ± 720.68, *p* < 0.001) after OPCAB than the patients with non-TXA. Therefore, the risk of total blood exposure [odds ratio (OR) = 0.50, 95% CI 0.47–0.54, *p* < 0.001] or blood component exposure (*p* < 0.001) was decreased significantly in the patients who administered TXA. The TXA dosage did not impact the patient survival, thromboembolic events, or blood management.

**Conclusions:**

The application of TXA was safe and provided blood control in patients with OPCAB, and the dosage did not affect these parameters.

## Introduction

Bleeding and blood infusions are common during coronary artery bypass graft (CABG) surgeries (1). Antifibrinolytic agents that have been used in patients who underwent cardiac surgery include aprotinin and the lysine analogs tranexamic acid (TXA). The TXA was revealed to have excellent hemostasis effects in cardiac surgeries without significant thromboembolic events (2). Cardiopulmonary bypass (CPB) initiated during cardiac surgery was demonstrated to activate clotting, exhaust coagulation factors, and cause platelet dysfunction and excessive fibrinolysis (3). Many clinical trials have demonstrated the effectiveness of TXA to maintain hemostasis in on-pump CABG (4, 5).

Although CPB avoidance during off-pump CABG (OPCAB) could reduce the blood exposure risk, hypothermia, acidosis, and tissue trauma still contributes to inadequate hemostasis during OPCAB (6). During OPCAB, serious trauma (sternotomy, internal mammary artery or saphenous vein graft harvesting, pericardiotomy, and heart manipulation) and heparin and protamine exposure activate coagulation by releasing tissue factors and activating extrinsic pathways (7). Therefore, blood transfusions are still needed for OPCAB, and complications after blood infusion have become one of the main concerns with OPCAB (8).

A greater activation level of fibrinogen and other acute-phase proteins has been observed in OPCAB compared with on-pump CABG, which may lead to higher thromboembolic event risk in OPCAB (9). Therefore, the safety profiles of TXA in OPCAB should be further considered.

Clinical studies on the application of TXA during OPCAB obtained good blood management results (10, 11), and less bleeding and fewer blood transfusions were observed after TXA administration during OPCAB (10–12). However, the study evidence lacked robustness because of the small patient population. In this study, the impact of TXA on both hemostasis and safety issues during OPCAB was revealed in a large retrospective cohort by propensity score matching (PSM).

## Materials and Methods

### Patient Population

**Figure 1:**
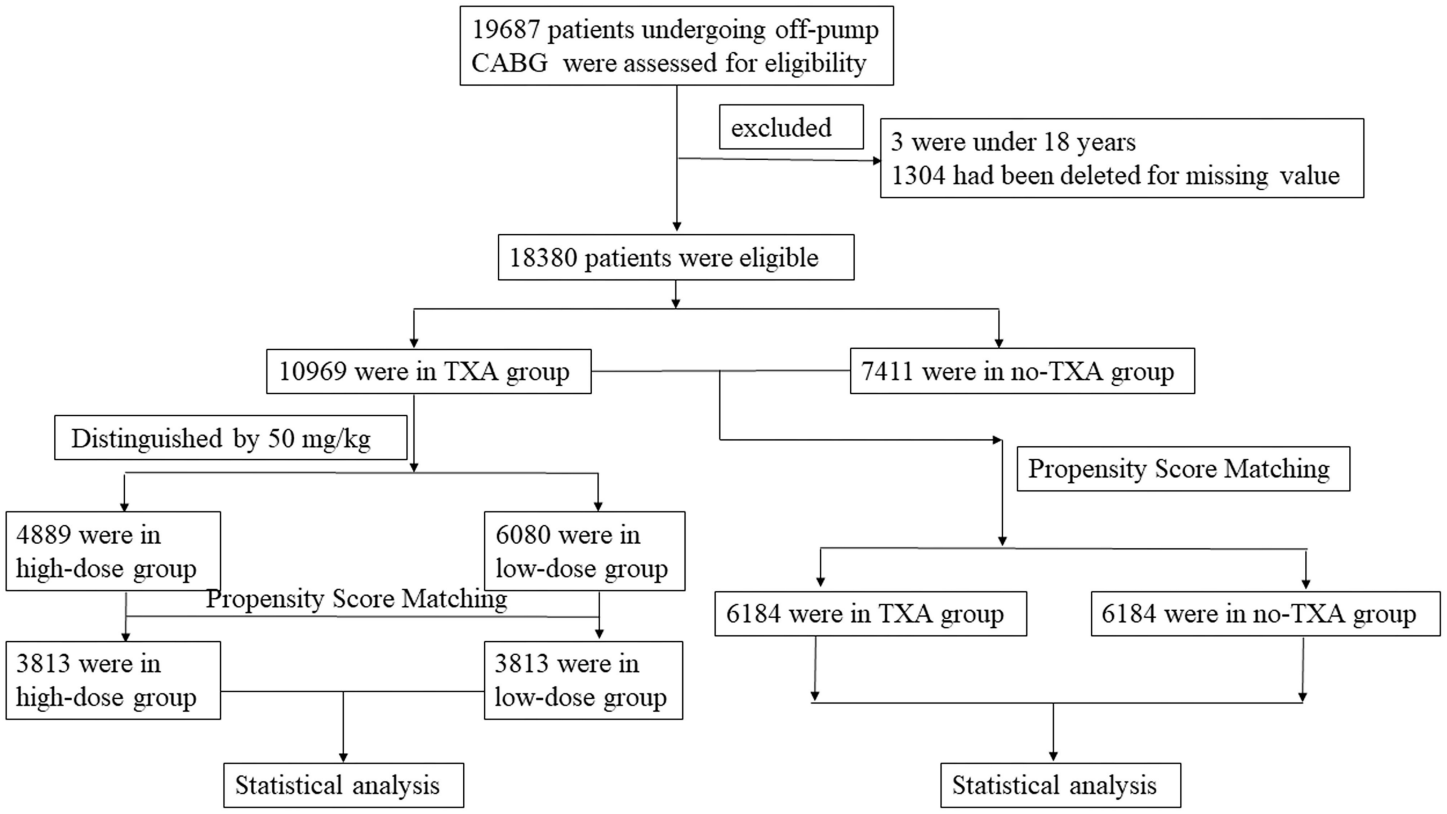
Flowchart of OPCAB patients with or without TXA administration. This study included 19,687 patients who underwent OPCAB. Patients under 18 years old or with missing values were excluded from this cohort. A total of 18,380 OPCAB patients were finally included in this study. Among them, 10,969 patients were in the TXA group and 7,411 patients were in the no-TXA group. There were 4,889 patients whose TXA dose was ≥50 mg/kg, and the remaining 6,080 patients had a TXA dose of <50 mg/kg. Propensity score matching was performed in the TXA and no-TXA groups and the high-dose and low-dose groups. A statistical analysis was then performed. OPCAB, off-pump coronary artery bypass grafts; TXA, tranexamic acid.

This study included 19,687 patients who underwent OPCAB from January 1, 2009 to December 31, 2019. Patients under 18 years of age and those with missing values were excluded from this cohort. Finally, 18,380 patients were enrolled in the statistical analysis (Figure 1). Among them, 10,969 patients with TXA administration during OPCAB were selected as the TXA group and 7,411 patients without TXA application during OPCAB were selected as the no-TXA group. According to the literature (13, 14), the cutoff TXA dose was set to 50 mg/kg. The high-dose TXA group included 4,889 patients with a TXA dose ≥50 mg/kg, and the low-dose TXA group included 6,080 patients with a TXA dose <50 mg/kg. Baseline and surgical information for the whole OPCAB cohort are shown in Tables 1, 2, while the TXA dose subgroup information is available in Supplementary Tables 1, 2. The study was carried out according to the Declaration of Helsinki 1964 and its subsequent amendments. The Medical Ethics Committee of Fuwai Hospital approved the protocol. All enrolled patients provided signed informed consent.

**Table 1:** Baseline information for OPCAB patients with or without TXA in the entire cohort before and after propensity score matching.

**Characteristics**	**Before matching**			**After matching**		
	**TXA group** **(***n*** = 10,969)**	**No TXA group** **(***n*** = 7,411)**	***p-*** **value**	**TXA group** **(***n*** = 6,184)**	**No TXA group** **(***n*** = 6,184)**	***p*** **-value**
Age (y), mean ± SD	61.65 ± 8.70	61.64 ± 8.90	0.912	61.55 ± 8.74	61.61 ± 8.87	0.664
BMI (kg/m^2^), mean ± SD	25.75 ± 3.08	25.76 ± 3.08	0.873	25.76 ± 3.14	25.77 ± 3.08	0.844
Male sex, *n* (%)	8,429 (76.8)	5,831 (78.7)	0.003	4,805 (77.7)	4,794 (77.5)	0.827
NYHA III-V, *n* (%)	3,448 (31.4)	1,918 (25.9)	<0.001	1,679 (27.2)	1,765 (28.5)	0.076
LV dysfunction (ejection fraction < 40%), *n* (%)	375 (3.4)	179 (2.4)	<0.001	173 (2.8)	170 (2.7)	0.912
**Pre-existing medical conditions**, ***n*** **(%)**						
Insulin dependent diabetes	1,364 (12.4)	759 (10.2)	<0.001	681 (11.0)	696 (11.3)	0.684
Hyperlipidemia	7,972 (72.7)	4,629 (62.5)	<0.001	4,135 (66.9)	4,148 (67.1)	0.808
Hypertension	7,023 (64.0)	4,535 (61.2)	<0.001	3,893 (63.0)	3,880 (62.7)	0.821
Chronic kidney disease	740 (6.7)	451 (6.1)	0.074	407 (6.6)	382 (6.2)	0.375
COPD	147 (1.3)	127 (1.7)	0.040	87 (1.4)	102 (1.6)	0.303
Peripheral vascular disease	1,223 (11.1)	690 (9.3)	<0.001	606 (9.8)	626 (10.1)	0.566
Cerebrovascular accident	1,572 (14.3)	936 (12.6)	0.001	813 (13.1)	823 (13.3)	0.810
Previous cardiac surgery	358 (3.3)	179 (2.4)	0.001	178 (2.9)	168 (2.7)	0.619
Preoperative atrial fibrillation	196 (1.8)	113 (1.5)	0.175	97 (1.6)	97 (1.6)	1.000
Acute coronary syndrome	2,378 (21.7)	1,741 (23.5)	0.004	1,397 (22.6)	1,408 (22.8)	0.828
Left main stem disease	1,382 (12.6)	1,387 (18.7)	<0.001	961 (15.5)	959 (15.5)	0.979
Three-vessel disease	8,329 (75.9)	5,524 (74.5)	0.031	4,592 (74.3)	4,575 (74.0)	0.732
Pre-operative IABP	133 (1.2)	60 (0.3)	0.009	60 (1.0)	59 (1.0)	1.000
Time between CAG and operation <3 days	240 (2.2)	241 (3.3)	<0.001	148 (2.4)	171 (2.8)	0.202
No. of risk factors for bleeding			<0.001			0.429
0–1	7,430 (67.5)	5,192 (70.1)		4,261 (68.9)	4,251 (68.7)	
2–3	3,407 (31.1)	2,148 (29.0)		1,846 (29.9)	1,872 (30.3)	
4–5	159 (1.4)	71 (1.0)		77 (1.2)	61 (1.0)	
**Pre-operative medications**, ***n*** **(%)**						
Aspirin within last 5 days	1,738 (15.8)	939 (12.7)	<0.001	875 (14.1)	874 (14.1)	1.000
Clopidogrel within last 5 days	1,950 (16.2)	1,203 (16.2)	0.006	1,079 (17.4)	1,072 (17.3)	0.883
Ticagrelor within last 5 days	106 (1.0)	42 (0.6)	0.003	46 (0.7)	40 (0.6)	0.590
LWMH within 24 h	2,822 (25.7)	1,812 (24.5)	0.051	1,506 (24.4)	1,504 (24.3)	0.983
ACEI or ARB	3,989 (36.4)	3,112 (42.0)	<0.001	2,488 (40.2)	2,479 (40.1)	0.881
Nitrate	10,577 (96.4)	7,143 (96.4)	0.879	5,951 (96.2)	5,943 (96.1)	0.744
Beta blocker	9,309 (84.9)	6,405 (86.4)	0.003	5,288 (85.5)	5,274 (85.3)	0.737
Calcium-channel blocker	2,443 (22.3)	1,722 (23.2)	0.126	1,409 (22.8)	1,381 (22.3)	0.560
Statin	9,094 (82.9)	5,588 (7.4)	<0.001	4,852 (78.5)	4,810 (77.8)	0.357
**Pre-operative laboratory tests**						
eGFR (ml/min/1.73 m^2^), mean ± SD	91.13 ± 21.64	91.73 ± 22.13	0.071	91.84 ± 21.88	91.51 ± 21.97	0.393
Hb male/female < 130/120 g/L), *n* (%)	2,591 (23.6)	1,716 (23.2)	0.464	1,396 (22.6)	1,471 (23.8)	0.357
Propensity score, mean ± SD	0.64 ± 0.14	0.57 ± 0.14	<0.001	0.57 ± 0.14	0.57 ± 0.14	<0.001

*TXA, tranexamic acid; BMI, body mass index; NYHA, New York Heart Association (classification); LV, left ventricle; COPD, chronic obstructive pulmonary disease; IABP, intra-aortic balloon pump; CAG, coronary angiography; LMW, low-molecular-weight heparin; ACEI, angiotensin-converting enzyme inhibitor; ARB, angiotensin-receptor blocker; eGFR, estimated glomerular filtration rate; Hb, hemoglobin*.

**Table 2:** Surgical and other perioperative characteristics of the patients in the tranexamic acid and no tranexamic acid groups before and after propensity score matching.

**Surgery**	**Before matching**			**After matching**		
	**TXA group** **(***n*** = 10,969)**	**No TXA group** **(***n*** = 7,411)**	***p-*** **value**	**TXA group** **(***n*** = 6,184)**	**No TXA group** **(***n*** = 6,184)**	***p-*** **value**
CABGs by experienced surgeons (≥100 CABGs/year), *n* (%)	8,961 (81.7)	6,315 (85.2)	<0.001	5,163 (83.5)	5,196 (84.0)	0.399
Operation year (2009–2014), *n* (%)	3,750 (34.2)	4,638 (62.6)	<0.001	3,417 (55.3)	3,413 (55.2)	0.867
High risk operation, *n* (%)	608 (5.5)	343 (4.6)	0.006	324 (5.2)	314 (5.1)	0.713
Emergent surgery, *n* (%)	257 (2.3)	166 (2.2)	0.648	151 (2.4)	147 (2.4)	0.860
Elective, *n* (%)	10,712 (97.7)	7,245 (97.8)	0.648	6,033 (97.6)	6,037 (97.6)	0.860
Isolated CABG, *n* (%)	10,899 (99.4)	7,354 (99.2)	0.293	6,150 (99.5)	6,131 (99.1)	0.054
**Operative data**
Heparin neutralization ratio, mean ± SD	1.01 ± 0.39	1.08 ± 0.35	<0.001	1.06 ± 0.41	1.06 ± 0.35	0.541
Left internal mammary artery, *n* (%)	10,146 (92.5)	6,768 (91.3)	0.004	5,682 (91.9)	5,692 (92.0)	0.763
Distal anastomoses (number), mean ± SD	3.14 ± 1.02	3.17 ± 0.97	<0.001	3.17 ± 1.02	3.16 ± 0.98	0.477
Duration of surgery (min), mean ± SD	250.26 ± 57.13	258.97 ± 70.93	<0.001	253.95 ± 51.67	253.88 ± 57.10	0.941

*TXA, tranexamic acid; CABG, coronary artery bypass graft; high risk: previous cardiac surgery, emergent surgery, CABG combined with valve operation and CABG combined with aortic or arch operation; open chamber: CABG combined with valve surgery or aortic surgery or aneurysm resection*.

### Operation Details

All patients received OPCAB surgery with or without TXA administration during the operation according to the anesthesiologists' preference. A 1 g dose of TXA was administered 30 min before skin incision at 2 g/h and continued at 200–800 mg/h during the entire surgical procedure. The continuous infusion protocol was based on the patients' weight and the anesthesiologists' judgment. Heparin (200 IU/kg) was administered before the transection of the internal mammary artery to maintain the activated clotting time (ACT) at 300 s. After the proximal anastomosis was finished and no major hemorrhage was observed, protamine was administered at a ratio of 1 mg protamine to 100 IU heparin. Additional protamine was administered during chest closure or in the intensive care unit (ICU) according to the immediate ACT results and the surgeons', anesthesiologists', and ICU physicians' judgment.

### Outcome Measurement

Hospital death and thromboembolic events [such as perioperative myocardial infarction (PMI), stroke, acute kidney injury (AKI), and pulmonary embolism] were defined as primary endpoints. The secondary endpoints consisted of blood loss and blood exposure after OPCAB. Blood loss after surgery included blood loss at 24 and 48 h and total blood loss after OPCAB. Blood exposure after OPCAB included red blood cell (RBC) exposure, fresh frozen plasma (FFP) exposure, and platelet (PLT) exposure after surgery.

### Statistical Analysis

For continuous variables, the mean ± SD was used for normally distributed variables while the median and interquartile range (IQR) were used for non-normally distributed variables. The categorical variables were expressed as numbers and percentages. The statistical significance of the baseline and perioperative variables between the TXA and no-TXA groups or between the high-dose and low-dose groups before PSM was calculated by Student's *t*-test for normal distribution and Mann-Whitney U test for non-normal distribution of continuous variables. The significant differences in categorical variables between groups were assessed by the χ^2^-test or Fisher's exact-test before PSM. To calculate the statistical significance of the baseline and perioperative information after PSM, the paired Student's *t*-test was used for normally distributed variables, the Wilcoxon rank test was used for non-normally distributed variables, while the paired χ^2^-test was calculated for categorical variables. Conditional logistic regression was selected for the outcome analysis after PSM. The odds ratio (OR) and 95% CIs were used for binary outcome variables.

The 1:1 matching propensity score was used for patients between the TXA and no-TXA groups throughout the entire OPCAB cohort or between the high-dose and low-dose groups in the TXA subset. In addition, a caliper width of 0.01 and the nearest-neighbor matching method without replacement were used for PSM. Thirty-two covariates were selected for PSM in accordance with the clinical and statistical significance (*p* < 0.05): age; body mass index; sex (male); left ventricular dysfunction; insulin-dependent diabetes; hyperlipidemia; hypertension; chronic kidney disease (CKD); peripheral vascular disease; cerebrovascular accident; previous cardiac surgery; acute coronary syndrome; left main stem disease; three-vessel disease; pre-operative intra-aortic balloon pump; time between coronary angiography and operation <3 days; risk factors for bleeding; use of aspirin, clopidogrel, or ticagrelor within 5 days before surgery; low-molecular-weight heparin within 24 h pre-operatively; angiotensin-converting enzyme inhibitors or angiotensin-receptor blockers; use of nitrates, beta-blockers, calcium-channel blockers, and statins; the surgeons' experience (≥100 CABGs/year); operation year (years from 2009 to 2014 and 2015 to 2019 were divided as binary covariates); emergency surgery; heparin neutralization ratio (HNR); and left internal mammary artery duration of surgery (min).

**Figure 2:**
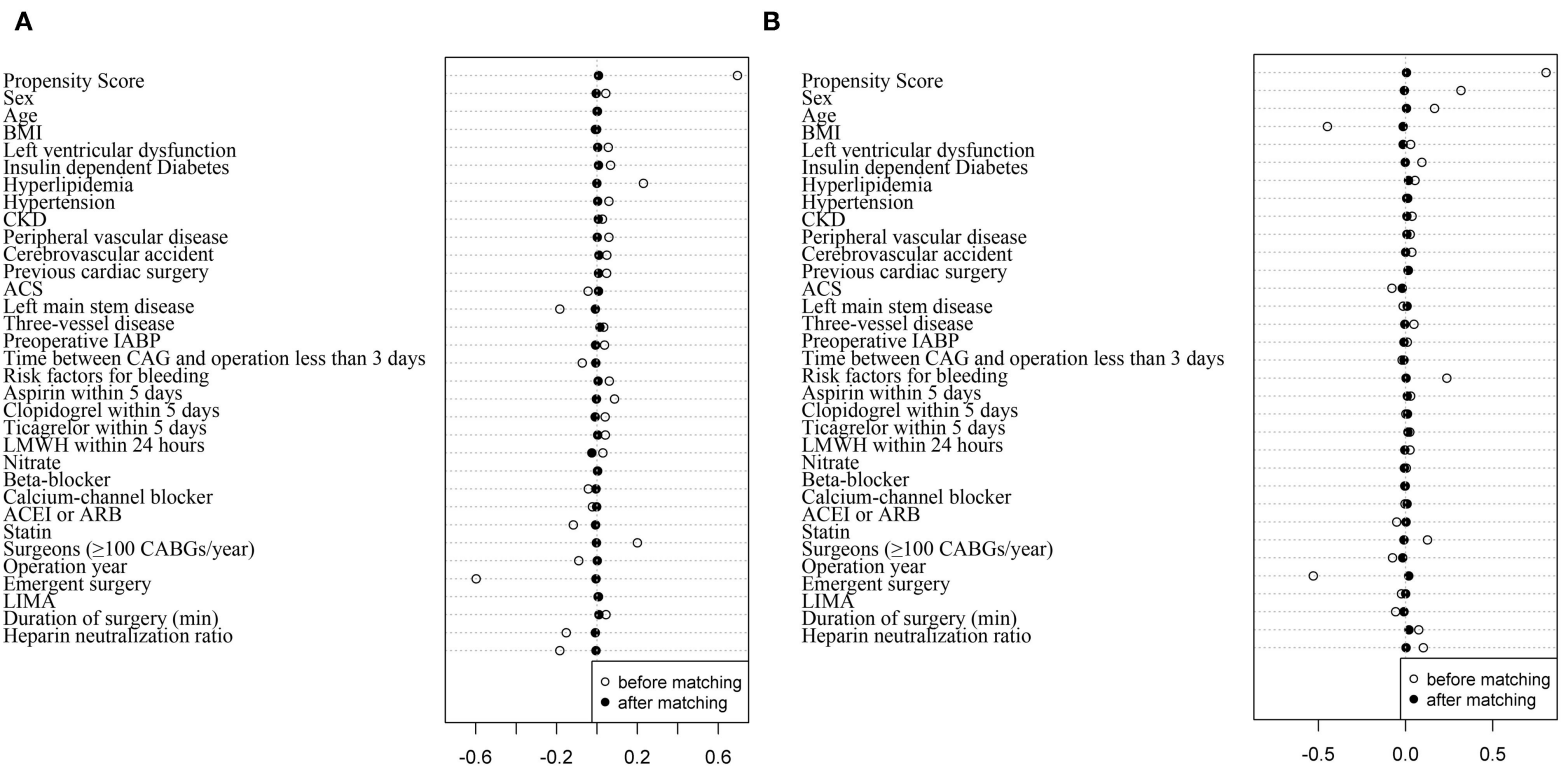
Propensity score matching in patients with OPCAB between the TXA and no-TXA groups and the high-dose and low-dose subgroups. **(A)** The propensity score matched well between the TXA and no-TXA groups. The standardized difference was <0.1 in all 32 covariates. **(B)** The propensity score matched well between the high-dose and low-dose subgroups. The standardized difference was <0.1 in all 32 covariates.

After PSM, 6,184 patients in the TXA group and 6,184 patients in the no-TXA group were matched based on a standardized difference <0.1 (Figure 2A). In the TXA subset, 3,813 patients in the high-dose group and 3,813 patients in the low-dose group were matched well (standardized difference <0.1; Figure 2B). The balance was well-maintained in all OPCAB patients (Tables 1, 2) and in the TXA dose subgroup (Supplementary Tables 1, 2).

A sensitivity analysis was performed for all patients with OPCAB and the TXA subgroups by a binary logistic analysis using the “enter” method. Thirty-two variables and TXA or TXA dose were chosen as covariates. Binary outcome variables were selected as dependent variables. The ORs and 95% CIs were used to express the statistical results. Statistical significance was expressed as *p* < 0.05.

In this study, International Normalized Ratio [INR] values were missing for 1,304 patients (6.6%); thus, the information for these patients was excluded from the following statistical analysis. A value of “1” was input for the missing INR values based on the normal neutralization as referenced above. Then, 19,684 patients with OPCAB in the entire OPCAB cohort and 11,206 patients in the TXA subgroup were included in the sensitivity analysis. The details of the sensitivity analysis were the same as previously described.

All statistical analyses were carried out with IBM SPSS Statistics for Windows, version 22.0 (IBM Corp., Armonk, NY, USA).

## Results

### Baseline Information

The demographic, medical, and surgical characteristics of the patients with OPCAB presented significant differences between the TXA and no-TXA groups (*p* < 0.05), whereas after PSM, significant differences were not observed among the variables between these two groups (Tables 1, 2). In patients with TXA administration, certain baseline variables were significantly different between the high-dose and low-dose groups (*p* < 0.05). The median TXA dose in the high-dose (≥50 mg/kg) group was 66.67 mg/kg, with an IQR from 57.69 to 75.76 mg/kg. The median TXA dose in the low-dose group (<50 mg/kg) was 39.68 mg/kg, with an IQR from 34.72 to 43.87 mg/kg. After PSM, differences were observed in the New York Heart Association III–V (34.7% in the TXA group vs. 31.8% in the no-TXA group, *p* = 0.009) and chronic obstructive pulmonary disease parameters (1.6% in the TXA group vs. 1.0% in the no-TXA group, *p* = 0.042; Supplementary Tables 1, 2). Therefore, the PSM results remained well-balanced between the TXA and no-TXA groups or the high-dose group and the low-dose group.

### Primary and Secondary Outcomes After PSM in OPCAB Patients With or Without TXA Application

Tranexamic acid administration increased the risk of death and thromboembolic events (primary endpoint) by 1.17-fold (OR = 1.17, 95% CI 1.01–1.36, *p* = 0.039). However, TXA administration did not increase the risk of hospital death, PMI, stroke, AKI, or pulmonary embolism (Table 3). After TXA administration, the risk of reoperation because of major hemorrhage or cardiac tamponade was decreased by 0.28-fold (OR = 0.72, 95% CI 0.53–0.96, *p* = 0.027; Table 3), and these patients also showed less blood loss at 24 h (478.32 ± 276.41 vs. 641.28 ± 295.09, *p* < 0.001) and 48 h (730.59 ± 358.55 vs. 915.24 ± 390.13, *p* < 0.001) and less total blood loss (989.00 ± 680.43 vs. 1,220.01 ± 720.68, *p* < 0.001) after OPCAB than patients with non-TXA (*p* < 0.001; Table 3). Therefore, the risk of total blood exposure (OR = 0.50, 95% CI 0.47–0.54, *p* < 0.001) or RBC (OR = 0.55, 95% CI 0.51–0.60, *p* < 0.001), FFP (OR = 0.39, 95% CI 0.36–0.43, *p* < 0.001), and PLT transfusion exposure (OR = 0.60, 95% CI 0.41–0.88, *p* < 0.001) was decreased significantly with TXA administration (Table 3). However, the length of hospital stay or ICU stay time was not influenced by TXA administration. Differences in any cause mortality within 30 days after hospitalization or seizure were not observed between the two groups (Table 3).

**Table 3:** Adjusted odds ratios in patients with OPCAB for primary and secondary endpoints between the TXA group and no-TXA group by PSM.

**Outcomes**	**TXA group** **(***n*** = 6,184)**	**No-TXA group** **(***n*** = 6,184)**	**OR (95%CI)**	***p-*** **value**
**Primary endpoint**, ***n*** **(%)**	367 (5.9)	313 (5.1)	1.17 (1.01–1.36)	0.039
Hospital death	9 (0.1)	7 (0.1)	1.29 (0.48–3.45)	0.618
Myocardial infarction	177 (2.9)	143 (2.3)	1.24 (0.99–1.54)	0.058
Stroke	47 (0.8)	34 (0.5)	1.38 (0.89–2.15)	0.15
Acute renal injury	159 (2.6)	139 (2.2)	1.14 (0.91–1.44)	0.247
Pulmonary embolism	7 (0.1)	6 (0.1)	1.17 (0.39–3.47)	0.782
**Blood loss after operation**
Reoperation due to major hemorrhage or cardiac tamponade, *n* (%)	76 (1.2)	106 (1.7)	0.72 (0.53–0.96)	0.027
Blood loss in 24 h after surgery (mL), mean ± SD	478.32 ± 276.41	641.28 ± 295.09		<0.001
Blood loss in 48 h after surgery (ml), mean ± SD	730.59 ± 358.55	915.24 ± 390.13		<0.001
Total blood loss after surgery (ml), mean ± SD	989.00 ± 680.43	1,220.01 ± 720.68		<0.001
**Blood transfusion after surgery**, ***n*** **(%)**
Blood transfusion	1,132 (18.3)	2,267 (36.7)	0.50 (0.47–0.54)	<0.001
RBC	813 (13.1)	1,472 (23.8)	0.55 (0.51–0.60)	<0.001
FFP	579 (9.4)	1,481 (23.9)	0.39 (0.36–0.43)	<0.001
PLT	42 (0.7)	70 (1.1)	0.60 (0.41–0.88)	<0.001
**Post-operative course**
Intensive care (h), median (IQR)	48 (IQR 24–96)	48 (IQR 24–96)		0.866
Hospital stay (d), mean ± SD	17.21 ± 7.83	17.34 ± 8.08		0.393
**Adverse events after surgery**, ***n*** **(%)**
Death from any cause within 30 days	19 (0.3)	14 (0.2)	1.36 (0.68–2.71)	0.383
Seizure	6 (0.1)	8 (0.1)	0.75 (0.26–2.16)	0.594

*TXA, tranexamic acid; RBC, red blood cell; FFP, fresh frozen plasma; PLT, platelet; OR, odds ratio; CI, confidence interval; IQR, interquartile range; SD, standard deviation*.

### Sensitivity Analysis in OPCAB Patients With or Without TXA Application

The sensitivity analysis revealed that the risk of the primary endpoint was not associated with TXA (Supplementary Table 3). A reduced risk of reoperation after surgery was observed in the TXA group compared to the no-TXA group (OR = 0.67, 95% CI 0.52–0.87, *p* = 0.003). The risk of blood exposure (OR = 0.36, 95% CI 0.34–0.39, *p* < 0.001) or blood component exposure was also decreased by TXA application (Supplementary Table 3). The sensitivity analysis after the imputation of missing values also presented similar results as the sensitivity referred to above (Supplementary Table 3).

### Primary and Secondary Outcomes in OPCAB Patients With High- or Low-Dose TXA

After PSM, the high-dose TXA group was not associated with the risk of the primary endpoint or each component (*p* > 0.05; Supplementary Table 4). Moreover, the TXA dose was not associated with either blood loss or blood exposure (*p* > 0.05; Supplementary Table 4). Differences were not observed in the length of hospital stay, ICU stays, death at 30 days, or seizures between the two groups (*p* > 0.05; Supplementary Table 4). The sensitivity analysis of the TXA group with or without imputation of missing values revealed similar results as the PSM analysis (Supplementary Table 5).

## Discussion

This study used PSM to evaluate the effects of TXA application in patients with OPCAB in a large cohort. Blood loss and blood exposure after surgery were significantly reduced by TXA administration.

Although off-pump CABG presents a lower requirement for blood transfusion and has a lower hemorrhage-related re-exploration rate than on-pump CABG (8), perioperative hemorrhage complications and the consequent blood infusion are still one of the main concerns after OPCAB (15). The blood loss and blood exposure after TXA application were both reduced in this study, which was also revealed in literature published before. TXA, as an antifibrinolytic agent, was also demonstrated to control bleeding and decrease blood infusion in OPCAB in some small randomized studies and meta-analyses (11, 16–18). A randomized control trial (RCT) by Wang et al. (17) indicated that included 231 patients with OPCAB revealed that TXA reduced post-operative chest tube drainage and the requirement for RBC and FFP transfusion at 24 h after surgery. In a 348 patient cohort study by Weingarten et al. (16), post-operative chest tube drainage was significantly reduced in patients who received TXA at 24 h after OPCAB compared with the non-TXA group, although differences in blood transfusion were not observed. The two referenced meta-analyses also revealed a definite reduction in blood loss and blood exposure in patients with OPCAB after TXA administration (11, 18). The evidence cited above indicates that TXA supported effective blood management for OPCAB surgery.

In this study, TXA administration was not associated with hospital death, PMI, stroke, AKI, or pulmonary embolism. Additionally, TXA did not increase the risk of post-operative adverse events. In the TXA dosage analysis, high-dose TXA was not associated with the primary endpoint or the secondary endpoints. The dosage also did not impact the risk of adverse events.

Our results were the same as the literature reported. In a meta-analysis of eight clinical trials on patients with OPCAB by Dai et al., associations between TXA and myocardial infarction, stroke, or pulmonary embolism were not observed (18). In another meta-analysis of 15 RCTs (11), 1,250 patients who underwent OPCAB were analyzed, and the results showed that TXA was not associated with post-operative death or thrombotic events. Although a greater activation level of fibrinogen and other acute-phase proteins was observed in OPCAB compared with on-pump CABG (9), the current results suggested that TXA application during OPCAB surgeries presented a negligible risk of hypercoagulation.

According to the literature (13, 14, 19), the cutoff TXA dose in this study was set as 50 mg/kg. Usually, TXA was administered at a dose of 30–100 mg/kg for a 4-h procedure (5, 20, 21). A TXA dosage ranging from 61 to 259 mg/kg could increase the incidence of clinical seizures after cardiac surgery (22). Myles et al. decreased the TXA dose from an initial 100 to 50 mg/kg to avoid TXA side effects, such as seizure and stroke, in an Aspirin and Tranexamic Acid for Coronary Artery Surgery (ATACAS) clinical trial (19). In a meta-analysis of 49 RCT studies with 10,591 patients who underwent cardiac surgery, a dose of TXA <50 mg/kg was defined as the low-dose group (13). Moreover, the results revealed that low-dose intravenous infusion was the most preferred delivery method and presented similar effectiveness as a high-dose regimen in reducing the transfusion rate without increasing the risk of seizures. Therefore, this study chose 50 mg/kg as the cutoff dose of TXA.

The seizure risk with TXA or TXA dosage in the present study was consistent with the study by Hulde et al., which included 2,249 PSM pairs of patients who underwent OPCAB with or without TXA administration. Differences were not observed in the risk of seizure, with values of 0.5 and 0.3% in the TXA and non-TXA groups, respectively (*p* = 0.36). Moreover, we had a lower risk of seizure in the TXA, no-TXA, and high-dose or low-dose group (0.1%). In the ATACAS study by Myles et al., the seizure risk was 0.7% in the TXA 100 mg/kg group vs. 0% in the no-TXA group and 0.5% in the TXA 50 mg/kg group vs. 0.1% in the no-TXA group. Although the TXA group had a higher risk than the no-TXA group at 100 or 50 mg/kg, the low-dose TXA decreased the seizure rate from 0.7 to 0.5%. Sharma et al. (23) found that the median dose of TXA in the seizure group was 100 mg/kg after analyzing 10,797 patients who underwent cardiac surgery by CPB. Both studies suggested that the administration of the high dose of TXA, particularly in doses exceeding 100 mg/kg, should be weighed against the risk of post-operative seizures. The median high-dose in this study was 66.67 mg/kg, while the low-dose was 39.68 mg/kg, both of which did not exceed 100 mg/kg. In the ATACAS study, on-pump CABG surgeries accounted for 97 and 96.8% in the TXA and no-TXA groups, respectively; therefore, the risk of seizure could primarily be explained by the inclusion of on-pump patients. TXA can cross a compromised blood-brain barrier (BBB), and it then binds to c-aminobutyric acid type A receptors, resulting in reduced inhibitory activity and increased neuronal excitation, thereby increasing the risk of seizure (24). These findings easily explain why cardiac surgery with CPB could compromise the BBB and induce seizures. However, patients with OPCAB could benefit from the lack of CPB when TXA is used. We concluded that the TXA application in OPCAB did not impact seizure risk.

Jerath et al. revealed that renal dysfunction could also influence the TXA concentration, which remained elevated above the therapeutic threshold for ~12 h in high-risk cardiac surgeries (25). Seizures mainly occurred in CKD stages III–V, which was also noticed in this study. CKD and high-risk surgeries were well balanced (Tables 1, 2). Therefore, the TXA dosage strategy should take CKD under consideration.

Although a large cohort of over 18,000 patients who underwent OPCAB surgery was included in this study, certain limitations were still observed. First, this was a retrospective study spanning 11 years; therefore, we could not obtain the intact baseline information needed for this analysis. Second, although PSM was chosen to simulate an RCT, potential cofounding covariates may have been present. Therefore, a large RCT clinical trial is needed for further study. Third, the numbers of anesthesiologists participated in over 18,000 OPCAB surgeries during the 11-year period, which gave us the difficulty to explore the reason for the use or no-use of TXA during surgeries. Finally, the definition of PMI in this study was according to the Society for Cardiovascular Angiography and Interventions (SCAI) (26), which was primarily based on the Troponin I value. The lack of electrocardiography and other imaging data made our inability to use the universal definition of PMI (27).

In conclusion, TXA administration did not increase the risk of hospital death, thromboembolic events, or seizures in patients who underwent OPCAB surgery. Moreover, TXA administration could decrease blood loss and prevent blood exposure, and differences were not observed between the high-dose and low-dose TXA groups in terms of adverse events and blood conservation.

## Data Availability Statement

The original contributions presented in the study are included in the article/Supplementary Material, further inquiries can be directed to the corresponding authors.

## Ethics Statement

The studies involving human participants were reviewed and approved by the Medical Ethics Committee of the Fuwai Hospital. The patients/participants provided their written informed consent to participate in this study.

## Author Contributions

EW, SY, and SH contributed to the study design. EW, XY, WC, and XZ contributed to the data collection. EW and YW contributed to the statistical analyses. EW contributed to the manuscript draft. SY and SH contributed to the final revision. All authors contributed to the article and approved the submitted version.

## Funding

Support was provided by the National Clinical Research Center of Cardiovascular Diseases, Fuwai Hospital, the Chinese Academy of Medical Sciences (Grant No. NCRC2020014), and the National Natural Science Foundation of China (Grant No. 81400242).

## Conflict of Interest

The authors declare that the research was conducted in the absence of any commercial or financial relationships that could be construed as a potential conflict of interest.

## Publisher's Note

All claims expressed in this article are solely those of the authors and do not necessarily represent those of their affiliated organizations, or those of the publisher, the editors and the reviewers. Any product that may be evaluated in this article, or claim that may be made by its manufacturer, is not guaranteed or endorsed by the publisher.

## Abbreviations

TXA: Tranexamic acid
CABG: Coronary artery bypass grafts
CPB: Cardiopulmonary bypass
OPCAB: Off-pump coronary artery bypass
PSM: Propensity score matching
ACT: Activated clotting time
ICU: Intensive care unit
PMI: Perioperative myocardial infarction
AKI: Acute kidney injury
RBC: Red blood cell
FFP: Fresh frozen plasma
PLT: Platelet
SD: standard deviation
IQR: Interquartile range
OR: Odds ratio
CI: Confidence interval
CKD: Chronic kidney disease
HNR: Heparin neutralization ratio
RCT: Randomized control trials
BBB: Blood–brain barrier.
