# Supplementary material for: Tranexamic Acid Administered During Off-Pump Coronary Artery Bypass Graft Surgeries Achieves Good Safety Effects and Hemostasis
Source: Front Cardiovasc Med. 2022 Feb 4;9:775760. doi: 10.3389/fcvm.2022.775760 (PMC8854353; doi:10.3389/fcvm.2022.775760)
Supplement: Supplementary file 1 [file Data_Sheet_1.docx]

Supplementary Material

1. Supplementary methods
   1. Estimated Glomerular filtration rates (eGFR): The eGFR was estimated using the simplified Modification of Diet in Renal Disease (MDRD) study equation as follows ^1^ eGFR=186.3×(serum creatinine in μmol per liter/88.4)^-1.154^×age^-0.203^×(0.742 if female).
   2. Chronic kidney disease (CKD): Kidney function is defined as normal with a GFR of more than 89 mL/min/1.73 m^2^ (stage I), minimally reduced with a GFR between 60 and 89 mL/min/1.73 m^2^ (stage II), moderately reduced with a GFR between 30 and 59 mL/min/1.73 m^2^ (stage III), severely reduced with a GFR between 15 and 29 mL/min/1.73 m^2^ (stage IV), and end-stage kidney failure with a GFR below 15 mL/min/ 1.73 m^2^ or renal replacement therapy (stage V). In this study, CKD was defined as kidney function≥ stage III ^2^.
   3. The risk factors for bleeding were older than 70 years, female sex, low-molecular-weight heparin or an antiplatelet drug less than 5 days before surgery, renal impairment (estimated glomerular filtration rate, <60 ml per minute), and insulin-dependent diabetes^3^.
   4. Open chamber operation: CABG with valve operation, CABG with ascending aortic or aortic arch operation and CABG with aneurysm resection were included in open chamber operation^4^.
   5. Primary endpoint:
      1. Hospital death: all-cause mortality after surgery during hospitalization.
      2. Perioperative myocardial infarction: is diagnosed by an Isolated elevation of CK-MB to ≥10×99th percentile upper reference limit (URL) or cTn (I or T) to ≥70×URL during the first 48 h following CABG surgery with or without ECG or imaging changes of MI^5,6^
      3. Stroke: A new-onset of neurological deficit symptoms secondary to cerebral infarction or hemorrhage on CT scan, and persisted for more than 24 h ^7^. A neurologist confirmed the diagnosis by reviewing the hospital records in all cases.
      4. Acute kidney injury(AKI): According to RIFLE (Risk, Injury, Failure, Loss, and End-stage kidney disease) criteria^8^, the definition of AKI was that the increased serum creatine×2 or GFR decrease > 50%.
      5. Pulmonary embolism: high probability VQ scan or documented on pulmonary angiogram.
   6. Secondary endpoint
      1. Blood transfusion: In our hospital, the packed red blood cell was transfused if the hemoglobin concentration below 7 g/dl during the cardiopulmonary bypass period and below 8 g/dl after operation^9^. The decision to transfuse fresh frozen plasma, platelets or surgical re-exploration was made under the consideration of blood loss, thromboelastogram ((TEG, Haemoscope Corporation, USA)) results, and patients’ hemodynamic stability by recovering unit physicians and surgeons together. The red blood cell, plasma or platelet transfusions were included in the blood transfusion.
2. Tables

| **Table S1 Characteristics of Patients in the high-dose and low-dose TXA subgroup** | | | | | | |
| --- | --- | --- | --- | --- | --- | --- |
| **Characteristics** | **Before matching** | | | **After matching** | | |
|  | **High-dose** (n=4889) | **Low-dose** | ***p* value** | **High-dose** (n=3813) | **Low-dose** (n=3813) | ***p* value** |
|  |  | (n=6080) |  |  |  |  |
| Age (y), mean ± SD | 62.43±8.44 | 61.03±8.86 | <0.001 | 61.77±8.52 | 61.74±8.65 | 0.872 |
| BMI (kg/m^2^), mean ± SD | 24.98±3.10 | 26.37±2.93 | <0.001 | 25.61±2.98 | 25.69±2.62 | 0.135 |
| Male sex, n (%) | 3358(68.7) | 5071(83.4) | <0.001 | 3005(78.7) | 2976(78.0) | 0.397 |
| NYHA III-V, n (%) | 1736(35.5) | 1712(28.2) | <0.001 | 1322(34.7) | 1213(31.8) | 0.009 |
| LV dysfunction (ejection fraction < 40 %), n (%) | 182(3.7) | 193(3.2) | 0.116 | 141(3.7) | 134(3.5) | 0.714 |
| **Preexisting medical conditions, n (%)** |  |  |  |  |  |  |
| Insulin dependent Diabetes | 696(14.2) | 668(11.0) | <0.001 | 494(13.0) | 489(12.8) | 0.893 |
| Hyperlipidemia | 3617(74.0) | 4355(71.6) | 0.006 | 2808(73.6) | 2830(74.2) | 0.584 |
| Hypertension | 3146(64.3) | 3877(63.8) | 0.528 | 2453(64.3) | 2450(64.3) | 0.962 |
| Chronic kidney disease | 356(7.3) | 384(6.3) | 0.045 | 271(7.1) | 248(6.5) | 0.309 |
| COPD | 78(1.6) | 69(1.1) | 0.037 | 60(1.6) | 39(1.0) | 0.042 |
| Peripheral vascular disease | 568(11.6) | 655(10.8) | 0.162 | 434(11.4) | 440(11.5) | 0.858 |
| Cerebrovascular accident | 735(15.0) | 837(13.8) | 0.060 | 560(14.7) | 524(13.7) | 0.250 |
| Previous cardiac surgery | 167(3.4) | 191(3.1) | 0.421 | 126(3.3) | 119(3.1) | 0.697 |
| Preoperative atrial fibrillation | 92(1.9) | 104(1.7) | 0.501 | 70(1.8) | 57(1.5） | 0.283 |
| Acute coronary syndrome | 974(19.9) | 1404(23.1) | <0.001 | 807(21.2) | 791(20.7) | 0.670 |
| Left main stem disease | 603(12.3) | 779(12.8) | 0.453 | 467(12.2) | 458(12.0) | 0.778 |
| Three-vessel disease | 3767(77.1) | 4562(75.0) | 0.014 | 2974(78.0) | 2997(78.6) | 0.541 |
| Preoperative IABP | 62(1.3) | 71(1.2) | 0.633 | 39(1.0) | 46(1.2) | 0.515 |
| Time between CAG and operation less than 3 days | 99(2.0) | 141(2.3) | 0.295 | 89(2.3) | 95(2.5) | 0.709 |
| No. of risk factors for bleeding |  |  | <0.001 |  |  | 0.500 |
| 0-1 | 2985(61.1) | 4418(72.7) |  | 2588(67.9) | 2588(67.9) |  |
| 2-3 | 1807(37.0) | 1600(26.3) |  | 1175(30.8) | 1176(30.8) |  |
| 4-5 | 97(2.0) | 62(1.0) |  | 50(1.3) | 49(1.3) |  |
| **Preoperative medications, n (%)** |  |  |  |  |  |  |
| Aspirin within last 5 days | 803(16.4) | 935(15.4) | 0.136 | 610(16.0) | 619(16.2) | 0.804 |
| Clopidogrel within last 5 days | 870(17.8) | 1080(17.8) | 0.965 | 667(17.5) | 673(17.7) | 0.881 |
| Ticagrelor within last 5 days | 54(1.1) | 52(0.9) | 0.185 | 47(1.2） | 38(1.0) | 0.386 |
| LWMH within 24 hours | 1288(26.3) | 1534(25.2) | 0.184 | 999(26.2) | 994(26.1) | 0.918 |
| ACEI or ARB | 1712(35.0) | 2277(37.5) | 0.008 | 1342(35.2) | 1328(34.8) | 0.759 |
| Nitrate | 4716(96.5) | 5861(96.4) | 0.859 | 3684(96.6) | 3684(96.6) | 1.000 |
| Beta-blocker | 4147(84.8) | 5162(84.9) | 0.910 | 3247(85.2) | 3255(85.4) | 0.823 |
| Calcium-channel blocker | 1085(22.2) | 1358(22.3) | 0.858 | 844(22.1) | 852(22.3) | 0.846 |
| Statin | 4173(85.4) | 4921(80.9) | <0.001 | 3225(84.6) | 3240(85.0) | 0.655 |
| **Preoperative laboratory tests** |  |  |  |  |  |  |
| eGFR (ml/min/1.73m^2^), mean ± SD | 91.13±21.89 | 91.14±21.45 | 0.990 | 90.98±21.69 | 90.73±21.38 | 0.608 |
| Hb male/female<130/120g/L), n (%) | 1293(26.4) | 1298(21.3) | <0.001 | 913(23.9) | 927(24.3) | 0.725 |
| Propensity Score,  mean ± SD | 0.53±0.18 | 0.46±0.15 | <0.001 | 0.46±0.15 | 0.46±0.15 | <0.001 |
| TXA, Tranexamic acid; BMI, Body mass index; NYHA, New York Heart Association (classification); LV, Left ventricle; COPD, Chronic obstructive pulmonary disease; IABP, Intra-aortic balloon pump; CAG, Coronary angiography; LMWH, low-molecular-weight heparin; ACEI Angiotensin converting enzyme inhibitor; ARB, Angiotensin-receptor blocker; eGFR, estimated Glomerular filtration rate; Hb, hemoglobin. | | | | | | |

| **Table S2. Surgical and Other Perioperative Characteristics of the Patients in the high-dose and low-dose TXA subgroups** | | | | | | |
| --- | --- | --- | --- | --- | --- | --- |
| **Surgery** | **Before matching** | | | **After matching** | | |
|  | **High-dose** (n=4889) | **Low-dose** (n=6080) | ***p* value** | **High-dose** (n=3813) | **Low-dose** (n=3813) | ***p* value** |
| CABGs by experienced surgeons (≥ 100 CABGs / year), n(%) | 3913(80.0) | 5048(83.0) | <0.001 | 3115(81.7) | 3126(82.0) | 0.766 |
| Operation year (2009-2014), n(%) | 1076(22.0) | 2674(44.0) | <0.001 | 1022(26.8) | 993(26.0) | 0.393 |
| High risk operation, n(%) | 269(5.5) | 339(5.6) | 0.867 | 202(5.3) | 207(5.4) | 0.836 |
| Emergent surgery, n(%) | 105(2.1) | 152(2.5) | 0.225 | 79(2.1) | 90(2.4) | 0.428 |
| Elective, n(%) | 4784(97.9) | 5928(97.5) | 0.225 | 3734(97.9) | 3723(97.6) | 0.428 |
| Isolated CABG, n(%) | 4854(99.3) | 6054(99.4) | 0.359 | 3789(99.4) | 3788(99.3) | 1.000 |
| **Operative data** |  |  |  |  |  |  |
| Heparin neutralization ratio, mean ± SD | 1.03±0.40 | 0.99±0.38 | <0.001 | 1.00±0.37 | 1.00±0.40 | 0.772 |
| Left internal mammary artery , n(%) | 4479(91.6) | 5667(93.2) | 0.002 | 3536(92.7) | 3547(93.0) | 0.656 |
| Distal anastomoses (number), mean ± SD | 3.14±1.03 | 3.13±1.01 | 0.813 | 3.17±1.02 | 3.16±1.01 | 0.682 |
| Duration of surgery( min), mean ± SD | 252.68±58.51 | 248.31±55.92 | <0.001 | 251.02±52.07 | 250.04±53.61 | 0.390 |
| TXA, Tranexamic acid; CABG, Coronary artery bypass graft; High risk: previous cardiac surgery, emergent surgery, CABG combined with valve operation and CABG combined with aortic or arch operation; Open chamber: CABG combined with valve surgery or aortic surgery or aneurysm resection. | | | | | | |

| **Table S3 Sensitivity analysis for primary and secondary endpoints between the TXA group and no TXA group in data with or without missing value imputation** | | | | |
| --- | --- | --- | --- | --- |
| **Outcomes** | **Data without missing value** | | **Data with missing value imputation** | |
|  | **OR(95% CI)** | ***p* value** | **OR(95% CI)** | ***p* value** |
| **Primary endpoint, n (%)** | 1.11(0.97-1.28) | 0.131 | 1.11(0.97-1.27) | 0.125 |
| Hospital death | 1.33(0.52-3.38) | 0.554 | 1.38(0.56-3.40) | 0.482 |
| Myocardial infarction | 1.21(0.99-1.48) | 0.062 | 1.16(0.96-1.41) | 0.122 |
| Stroke | 1.19(0.78-1.80) | 0.416 | 1.15(0.78-1.70) | 0.493 |
| Acute renal injury | 1.03(0.84-1.27) | 0.758 | 1.08(0.89-1.32) | 0.428 |
| Pulmonary embolism | 0.87(0.35-2.16) | 0.756 | 0.96(0.38-2.41) | 0.922 |
| **Blood loss after surgery** |  |  |  |  |
| Reoperation due to major hemorrhage or cardiac tamponade, n (%) | 0.67(0.52-0.87) | 0.003 | 0.68(0.53-0.89) | 0.004 |
| **Blood transfusion after surgery, n (%)** |  |  |  |  |
| Blood transfusion | 0.36(0.34-0.39) | <0.001 | 0.38(0.35-0.41) | <0.001 |
| RBC | 0.44(0.40-0.48) | <0.001 | 0.46(0.42-0.50) | <0.001 |
| FFP | 0.32(0.29-0.35) | <0.001 | 0.33(0.31-0.36) | <0.001 |
| PLT | 0.55(0.38-0.79) | 0.001 | 0.64(0.45-0.90) | 0.011 |
| **Adverse events after surgery, n (%)** |  |  |  |  |
| Death from any cause within 30 days | 1.25(0.67-2.32) | 0.492 | 1.13(0.83-2.01) | 0.689 |
| Seizure | 0.78(0.31-1.94) | 0.586 | 0.84(0.33-2.12) | 0.711 |
| TXA, Tranexamic acid; RBC, Red blood cell; FFP, fresh frozen plasma; PLT, platelet; OR, odds ratio; CI, confidence interval. | | | | |

| **Table S4 Adjust odds ratios in OPCAB patients for primary and secondary endpoints between the high-dose and low-dose TXA subgroups by PSM** | | | | |
| --- | --- | --- | --- | --- |
| **Outcome** | High-dose TXA group (n=3813) | Low-dose TXA group (n=3813) | OR 95% CI | *p* value |
| **Primary endpoint, n (%)** | 265(6.9) | 242(6.3) | 1.10(0.92-1.30) | 0.307 |
| Hospital death | 4(0.1) | 4(0.1) | 1.00(0.25-4.00) | 1 |
| Myocardial infarction | 145(3.8) | 137(3.6) | 1.06(0.84-1.34) | 0.634 |
| Stroke | 30(0.8) | 27(0.7) | 1.11(0.66-1.87) | 0.691 |
| Acute renal injury | 101(2.6) | 83(2.2) | 1.22(0.91-1.63) | 0.185 |
| Pulmonary embolism | 8(0.2) | 4(0.1) | 2.00(0.60-6.64) | 0.258 |
| **Blood loss after surgery** |  |  |  |  |
| Reoperation due to major hemorrhage or cardiac tamponade, n (%) | 46(1.2) | 40(1.0) | 1.15(0.75-1.76) | 0.518 |
| Blood loss in 24 hours after surgery (mL), mean ± SD | 420(IQR310-552) | 420(IQR310-560) |  | 0.202 |
| Blood loss in 48 hours after surgery (ml), mean ± SD | 650(IQR500-830) | 650(IQR500-850) |  | 0.107 |
| Total Blood loss after surgery(ml), mean ± SD | 830(IQR630-1130) | 830(IQR620-1140) |  | 0.819 |
| **Blood transfusion after surgery, n (%)** |  |  |  |  |
| Blood transfusion | 679(17.8) | 679(17.8) | 1.00(0.90-1.11) | 1 |
| RBC | 524(13.7) | 467(12.2) | 1.12(0.99-1.27) | 0.07 |
| FFP | 321(8.4) | 362(9.5) | 0.89(0.76-1.03) | 0.117 |
| PLT | 19(0.5) | 15(0.4) | 1.27(0.64-2.49) | 0.494 |
| **Postoperative course** |  |  |  |  |
| Intensive care (h), median (IQR) |  |  |  | 0.177 |
| Hospital stay (d),  mean ± SD | 16.94±7.14 | 16.85±7.62 |  | 0.607 |
| **Adverse events after surgery, n (%)** |  |  |  |  |
| Death from any cause within 30 days | 11(0.3) | 11(0.3) | 1.00(0.43-2.31) | 1 |
| Seizure | 3(0.1) | 3(0.1) | 1.00(0.20-5.00) | 1 |
| TXA, Tranexamic acid; RBC, Red blood cell; FFP, fresh frozen plasma; PLT, platelet; OR, odds ratio; CI, confidence interval; IQR, interquartile range; SD, standard deviation; | | | | |

| **Table S5. Sensitivity analysis for primary and secondary endpoints between the high-dose and low-dose TXA subgroups in data with or without missing value imputation** | | | | |
| --- | --- | --- | --- | --- |
| **Outcome** | **Data without missing value** | | **Data with missing value imputation** | |
|  | OR 95% CI | p value | OR 95% CI | p value |
| **Primary endpoint, n (%)** | 1.10(0.93-1.31) | 0.266 | 1.12(0.95-1.32) | 0.191 |
| Hospital death | 1.31(0.33-5.18) | 0.702 | 1.28(0.33-4.99) | 0.721 |
| Myocardial infarction | 1.05(0.84-1.31) | 0.668 | 1.02(0.82-1.27) | 0.866 |
| Stroke | 1.14(0.68-1.90) | 0.625 | 1.17(0.71-1.94) | 0.538 |
| Acute renal injury | 1.21(0.92-1.61) | 0.18 | 1.31(0.99-1.72) | 0.056 |
| Pulmonary embolism | 1.47(0.42-5.12) | 0.55 | 1.46(0.42-5.09) | 0.553 |
| **Blood loss after operation** |  |  |  |  |
| Reoperation due to major hemorrhage or cardiac tamponade, n (%) | 1.19(0.81-1.76) | 0.375 | 1.20(0.82-1.76) | 0.341 |
| **Blood transfusion after operation, n (%)** |  |  |  |  |
| Blood transfusion | 1.05(0.94-1.17) | 0.416 | 1.08(0.97-1.20) | 0.18 |
| RBC | 1.20(1.05-1.37) | 0.006 | 1.24(1.09-1.41) | 0.001 |
| FFP | 0.93(0.81-1.08) | 0.337 | 0.97(0.84-1.11) | 0.638 |
| PLT | 1.14(0.62-2.08) | 0.673 | 1.32(0.74-2.33) | 0.345 |
| **Adverse events after surgery, n (%)** |  |  |  |  |
| Death from any cause within 30 days | 1.11(0.51-2.44) | 0.793 | 1.06(0.48-2.31) | 0.891 |
| Seizure | 1.29(0.34-4.87) | 0.71 | 1.27(0.34-4.74) | 0.726 |
| TXA, Tranexamic acid; RBC, Red blood cell; FFP, fresh frozen plasma; PLT, platelet; OR, odds ratio; CI, confidence interval. | | | | |

1. References

1. Levey AS, Bosch JP, Lewis JB, Greene T, Rogers N, Roth D. A more accurate method to estimate glomerular filtration rate from serum creatinine: a new prediction equation. Modification of Diet in Renal Disease Study Group. *Ann Intern Med.* 1999;130(6):461-470.

2. Fox CS, Muntner P, Chen AY, Alexander KP, Roe MT, Wiviott SD. Short-term outcomes of acute myocardial infarction in patients with acute kidney injury: a report from the national cardiovascular data registry. *Circulation.* 2012;125(3):497-504.

3. Myles PS, Smith JA, Forbes A, et al. Tranexamic Acid in Patients Undergoing Coronary-Artery Surgery. *N Engl J Med.* 2017;376(2):136-148.

4. Waldow T, Szlapka M, Haferkorn M, Burger L, Plotze K, Matschke K. Prospective clinical trial on dosage optimizing of tranexamic acid in non-emergency cardiac surgery procedures. *Clin Hemorheol Microcirc.* 2013;55(4):457-468.

5. Moussa ID, Klein LW, Shah B, et al. Consideration of a new definition of clinically relevant myocardial infarction after coronary revascularization: an expert consensus document from the Society for Cardiovascular Angiography and Interventions (SCAI). *J Am Coll Cardiol.* 2013;62(17):1563-1570.

6. Thielmann M, Sharma V, Al-Attar N, et al. ESC Joint Working Groups on Cardiovascular Surgery and the Cellular Biology of the Heart Position Paper: Perioperative myocardial injury and infarction in patients undergoing coronary artery bypass graft surgery. *Eur Heart J.* 2017;38(31):2392-2407.

7. Min JJ, Nam K, Kim TK, et al. Relationship between early postoperative C-reactive protein elevation and long-term postoperative major adverse cardiovascular and cerebral events in patients undergoing off-pump coronary artery bypass graft surgery: a retrospective study. *Br J Anaesth.* 2014;113(3):391-401.

8. Bellomo R, Kellum JA, Ronco C. Defining and classifying acute renal failure: from advocacy to consensus and validation of the RIFLE criteria. *Intensive Care Med.* 2007;33(3):409-413.

9. Shi J, Zhou C, Liu S, et al. Outcome impact of different tranexamic acid regimens in cardiac surgery with cardiopulmonary bypass (OPTIMAL): Rationale, design, and study protocol of a multicenter randomized controlled trial. *Am Heart J.* 2020;222:147-156.
